# Genome-Wide SNP Markers Based on SLAF-Seq Uncover Breeding Traces in Rapeseed (*Brassica napus* L.)

**DOI:** 10.3389/fpls.2017.00648

**Published:** 2017-04-28

**Authors:** Qinghong Zhou, Can Zhou, Wei Zheng, Annaliese S. Mason, Shuying Fan, Caijun Wu, Donghui Fu, Yingjin Huang

**Affiliations:** ^1^Key Laboratory of Crop Physiology, Ecology and Genetic Breeding, Ministry of Education, Agronomy College, Jiangxi Agricultural UniversityNanchang, China; ^2^Jiangxi Institute of Red SoilJinxian, China; ^3^Plant Breeding Department, iFZ Research Centre for Biosystems, Land Use and Nutrition, Justus Liebig UniversityGiessen, Germany

**Keywords:** *Brassica napus* L., SLAF-seq, SNP loci, Population structure, LD analysis

## Abstract

Single Nucleotide Polymorphisms (SNPs) are the most abundant and richest form of genomic polymorphism, and hence make highly favorable markers for genetic map construction and genome-wide association studies. In this study, a total of 300 rapeseed accessions (278 representative of Chinese germplasm, plus 22 outgroup accessions of different origins and ecotypes) were collected and sequenced using Specific-Locus Amplified Fragment Sequencing (SLAF-seq) technology, obtaining 660.25M reads with an average sequencing depth of 6.27 × and a mean Q30 of 85.96%. Based on the 238,711 polymorphic SLAF tags a total of 1,197,282 SNPs were discovered, and a subset of 201,817 SNPs with minor allele frequency >0.05 and integrity >0.8 were selected. Of these, 30,877 were designated SNP “hotspots,” and 41 SNP-rich genomic regions could be delineated, with 100 genes associated with plant resistance, vernalization response, and signal transduction detected in these regions. Subsequent analysis of genetic diversity, linkage disequilibrium (LD), and population structure in the 300 accessions was carried out based on the 201,817 SNPs. Nine subpopulations were observed based on the population structure analysis. Hierarchical clustering and principal component analysis divided the 300 varieties roughly in accordance with their ecotype origins. However, spring-type varieties were intermingled with semi-winter type varieties, indicating frequent hybridization between spring and semi-winter ecotypes in China. In addition, LD decay across the whole genome averaged 299 kb when *r*^2^ = 0.1, but the LD decay in the A genome (43 kb) was much shorter than in the C genome (1,455 kb), supporting the targeted introgression of the A genome from progenitor species *B. rapa* into Chinese rapeseed. This study also lays the foundation for genetic analysis of important agronomic traits using this rapeseed population.

## Introduction

*Brassica napus* (AACC, 2*n* = 38) is an amphidiploid species originating from hybridization between *B*. *rapa* (AA, 2*n* = 20) and *B*. *oleracea* (CC, 2*n* = 18) within the past 10,000 years (Nagaharu, 1935). It is the world's second largest oilseed producing crop (behind soybean) and is planted in many countries worldwide, with an annual production of more than 60 million tons per year since 2011 (Shahzadi et al., 2015, https://apps.fas.usda.gov/psdonline/psdReport.aspx). Rapeseed oil is primarily an edible oil, but is also used as biofuel, as an industrial lubricant and as a base for polymer synthesis (Saeidnia and Gohari, 2012).

The domestication history of *B. napus* is rather short: only 400–500 years (Gómez-Campo and Prakash, 1999). However, targeted breeding in different climates and for different morphotypes has resulted in strong population structure (Rahman, 2013). Rapeseed germplasm worldwide can be differentiated into three ecotypes: winter (W), semi-winter (SW), and spring (S). These types result from long-term selection for low temperature vernalization and photoperiod sensitivity. Spring rapeseed with early flowering is mainly distributed in North America, Canada, and Australia; winter rapeseed with strict vernalization requirements is mainly distributed in Europe; and semi-winter rapeseed with moderate cold tolerance and vernalization requirements is planted primarily in the Yangtze valley of south China (Sun, 1946). In the past 20 years, the genetic diversity of the three ecotypes of *B. napus* has been widely studied by molecular marker technology (Diers and Osborn, 1994; Hasan et al., 2006; Qian et al., 2006, 2014).

Single nucleotide polymorphisms (SNPs) are DNA sequence variations that occur when a single nucleotide in the genome sequence is changed. SNPs are the most abundant form of genomic polymorphism, and SNP markers hence have higher density than any other marker type. Nowadays, it is possible to identify a large number of SNPs in a species quickly and efficiently via high-throughput DNA sequencing technologies. These have now been widely applied to develop massive genotyping arrays, which allow many more individuals in a species to be genotyped at an extremely high marker density in a fast, efficient, and highly reproducible way (Ganal et al., 2014). SNP markers have been used for a wide range of purposes in *Brassica*, including rapid identification of cultivars, QTL analysis, and construction of ultra-high-density genetic maps (Delourme et al., 2013). Moreover, SNPs provide valuable markers for the study of agronomic traits in crops via strategies such as genetic linkage mapping or association genetics (Han et al., 2016).

Specific locus amplified fragment sequencing (SLAF-seq) is a fast, accurate, highly efficient, and cost-effective method for developing large-scale SNP and InDel markers (Sun et al., 2013; Zhang et al., 2013). In this study, taking *B*. *napus* as the reference genome (Chalhoub et al., 2014) and using enzyme digestion techniques, a SLAF-seq library of specific size fragments of DNA was designed, sequences obtained, and polymorphic SLAF tags obtained by software alignment, finally resulting in identification of specific SNP sites. As an alternative approach for genotyping, SLAF-seq will be good to compare with the current sequencing-based technologies such as restriction-site associated DNA sequencing (RAD; Bus et al., 2012) and Diversity Arrays Technology sequencing (DArT-seq; Raman et al., 2014).

Population structure and linkage disequilibrium (LD) analysis are prerequisites for genome-wide studies of complex agronomic traits in a natural population (Ersoz et al., 2007). Population structure results from different allele frequencies between subgroups in a population, and suggests that members of every subgroup either have the same ancestors, or that they underwent the same environmental and/or artificial selection (Xiao et al., 2012). The existence of population structure and relative kinship in natural populations always results in a high level of spurious positives in association mapping (Yu et al., 2006). Many methods can be applied to remove and reduce the effects of these spurious positives, such as structure correlation analysis (Pritchard et al., 2000), Q + K mixed model systems (Yu et al., 2006), principal components analysis (PCA; Price et al., 2006), restricted maximum likelihood (REML; Stich and Melchinger, 2009) and efficient mixed-model association (EMMA; Kang et al., 2008). LD is the non-random recombination of alleles distributed on different loci (Gupta et al., 2005), and also is the prerequisite for association mapping, which determines the necessary marker density as well as the accuracy and choice of GWAS methods (Yu et al., 2006). Therefore, it is vital to understand LD levels and patterns in a population, and patterns of LD have been characterized in most major crop species. LD distances vary significantly between cross-pollinated and self-pollinated crops (Flint-Garcia et al., 2003). LD decays rapidly (within 1–5 kb) in diverse maize inbred lines (Yan et al., 2009), in cultivated sunflower (1.1 kb; Liu and Burke, 2006), and in wild grapevine (300 bp; Lijavetzky et al., 2007), whereas LD decays slowly in *Arabidopsis* (within 250 kb; Nordborg et al., 2002), in diverse rice lines (100–200 kb; McNally et al., 2009; Huang et al., 2010; Huang and Han, 2014), and in cultivated soybean (250 kb; Lam et al., 2010). In general, the LD decay distance in self-pollinated plants is much larger than in cross-pollinated species.

In this study, we carried out a genome-wide analysis of a set of 300 accessions representing eco-geographical diversity in China (278 lines) plus international varieties of *B. napus* (as outgroups). Each sample was sequenced using SLAF-seq, and genetic variation was analyzed by alignment to the *B. napus* reference genome (Chalhoub et al., 2014). Genetic diversity, population structure and linkage disequilibrium were evaluated with 201,817 newly developed genome-wide SNPs. Our research objectives were to (1) develop new *B. napus* SNPs and identify genetic variants using SLAF-seq; (2) assess the genetic diversity of our association mapping panel to deepen our understanding of the *B. napus* germplasm pool; and (3) investigate the population structure and the patterns of LD among the accessions, allowing us to deduce traces of breeding and distinct evolution in the A and C subgenomes. Our study also provides a valuable resource for further genome-wide association studies in *B. napus*, and paves the way for optimizing cross combinations, identifying loci closely related to agronomic traits, and exploiting rich allelic variation for marker-assisted breeding.

## Materials and methods

### Genotype selection and sampling

A set of 300 inbred rapeseed lines were included in the present study (Table S1), including 257 semi-winter types, 16 spring types, and 27 winter types. Germplasm was selected to represent variation in Chinese rapeseed (278 accessions), with an additional 22 accessions collected from Japan, Canada, the United States, and various European countries to represent exogenous rapeseed germplasm.

### Sample preparation and enzyme solution design

All accessions were collected from plants growing in the experimental fields at Jiangxi Agricultural University, Nanchang, China. Young healthy leaves were obtained from a single plant of each accession for DNA extraction using a modified cetyltrimethylammonium bromide (CTAB) method based on Murray and Thompson (1980). DNA concentration and quality of all samples was assessed with a Nanodrop 2000 UV-Vis spectrophotometer (NanoDrop, Wilmington, DE, USA). Quantified DNA was diluted to 100 ng·μl^−1^ for SLAF sequencing.

SLAF-seq success was predicted *in silico* using the 1.2 Gb *B. napus* reference genome of European winter homozygous oilseed cultivar “*Darmor-bzh*” (Chalhoub et al., 2014; http://www.genoscope.cns.fr/brassicanapus/data/). In order to acquire more than 250, 000 SLAF tags (defined as an enzyme fragment sequence of 314–414 bp) per genome, restriction enzyme combinations were tested and selected using *in silico* digestion prediction using the following criteria: (1) low percentage of restriction fragments comprising repeat sequences; (2) even distribution of restriction fragments across chromosomes; (3) simulated fragments align uniquely to the reference genome; and (4) high number of SLAF tags. Based on these four criteria, the restriction enzyme combination of *RsaI* and *HaeIII* (NEB, Ipswich, MA, USA) was selected.

### SLAF sequencing and data evaluation

Genomic DNA from each accession was digested with *RsaI* and *HaeIII* to obtain the SLAF tags, followed by fragment end reparation, dual-index paired-end adapter ligation, PCR amplification, and target fragment selection for SLAF library construction. Finally, the SLAF sequencing was carried out using an Illumina HiseqTM 2500 (Illumina, Inc; San Diego, CA, USA) at the Biomarker Technologies Corporation in Beijing.

The raw SLAF-seq data was processed for each sample using the software Dual-index (Kozich et al., 2013). After filtering out adapter reads, the sequencing quality was evaluated by calculating the guanine-cytosine (GC) content and Q30 (Q = –10^*^${\text{log}}_{10}^{e}$; indicating a 0.1% chance of an error and thus 99.9% confidence). Subsequently, all SLAF paired-end sample reads were clustered by the BLAT software according to sequence similarity (Kent, 2002). Polymorphic SLAF tags showed sequence polymorphisms between different accessions. High-quality SLAF tags were then mapped onto the reference genome of *B. napus* using the Burrows-Wheeler alignment tool (BWA) software (Li and Durbin, 2009), and the number of tags was counted.

### SNP loci identification

SNPs were identified based on the polymorphic SLAF tag information using the software programs GATK (McKenna et al., 2010) and SAMtools (Li et al., 2009): SNPs predicted from both methods were considered to be reliable. Ultimately, consistent SNPs were selected with the criteria of minor allele frequency (MAF) > 0.05 and integrity >80%.

### Analysis of SNP hotspots and SNP-rich regions between the three ecotypes

For each of the 201,187 SNPs identified, the mutation frequency per SNP was calculated, and SNP hotspots were defined as positions with SNP mutation frequency >0.8, such that most accessions differed from the *B. napus* reference genome sequence. In addition, the number of SNPs per 100 Kb along every chromosome was counted and sequenced, and the top 1% of regions in terms of number of SNPs present were identified as SNP-rich regions. SNP-rich regions were also calculated separately in each of the three rapeseed ecotypes.

### Analysis of genetic diversity and population structure

#### Calculation of genetic kinship between accessions

A total of 201,817 highly consistent SNPs were used to calculate pairwise kinship relationships among the 300 accessions using the software package SPAGeDi (Hardy and Vekemans, 2002). Negative kinship values between two accessions indicate less relationship than expected between them, and was corrected to 0 as proposed by Yu et al. (2006).

#### Phylogenetic tree construction and principal components analysis

Based on the 201,187 SNPs identified in this study, genetic distances were calculated between the 300 rapeseed accessions using the *p*-distance method (Jin and Nei, 1990). Phylogenetic trees were constructed using the MEGA5 software (Tamura et al., 2011), principal components analysis (PCA) was performed using GAPIT (Lipka et al., 2012), and the population structure of all accessions was analyzed with the software Admixture (Alexander et al., 2009).

#### Analysis of population structure and linkage disequilibrium (LD)

Based on the same set of SNPs, the number of subgroups (*K*) was predicted from 1 to 10, and the number of ancestors was determined according to the position of the minimum value, with error rate obtained from 5-fold cross-validation. Maximum likelihood estimates for the ancestry proportion from each *K* subgroup of each accession were calculated.

LD analysis proceeded based on the 201,817 SNPs using the software PLINK (specific parameters: MAF > 0.05, *r*^2^, ld-window 999999, ld-window- *r*^2^ 0, Purcell et al., 2007). LD in this population was assessed using the software package TASSEL 4.0 (Bradbury et al., 2007), and a cut-off value of *r*^2^ = 0.1 was set to estimate the extent of LD decay for each chromosome and across the A- and C-subgenomes respectively. The *r*^2^-value for a marker distance of 0 Kb was assumed to be 1.

#### Analysis of blocks based on linkage disequilibrium (LD)

The haplotype block structure in the 300 rapeseed accessions across the 201,187 SNPs was estimated with the HAPLOVIEW v4.2 software (Barrett et al., 2005). The number and size of haplotype blocks per chromosome was assessed.

## Results

### Assessment of experimental scheme

The restriction enzymes *RsaI* and *HaeIII* were selected based on *in silico* digestion prediction, and resulted in 281,218 predicted 314–414 bp SLAF tags with an average distance between SLAF tags of 4,267 bp (Table 1). Predicted SLAF tag distribution along each chromosome is shown in Figure S1A: tags were evenly distributed across the 19 chromosomes of *B. napus*. In total, 74.16% of paired-end reads had “normal” distances between both ends of 50 bp–1 Kb as predicted by BLAST, with a 97.58% digestion efficiency. Moreover, based on SLAF library construction and high-throughput sequencing, a total of 660.25 M reads were obtained to develop the SLAF tags, with a Q30 ratio of 85.96% and a GC content of 40.22% (Table S2).

**Table 1:** **Number of SLAF tags distributed on each chromosome of ***B. napus*****.

**Chromosome**	**Chromosome length (bp)**	**No. of expected SLAF**	**Average SLAF distance (bp)**	**No. of SLAF**	**Polymorphic SLAF**
A01	52,457,410	12,193	4,302	13,001	6,561
A02	53,983,291	12,551	4,301	13,899	7,531
A03	58,957,044	13,716	4,298	15,795	8,593
A04	48,341,214	11,029	4,383	9,726	5,708
A05	52,257,152	12,446	4,199	11,781	7,051
A06	53,585,940	12,458	4,301	13,546	7,659
A07	53,196,075	12,566	4,233	12,608	7,061
A08	48,151,495	11,184	4,305	10,103	5,236
A09	63,054,894	14,577	4,326	18,278	9,444
A10	46,587,781	11,118	4,190	9,764	5,898
C01	68,018,871	15,840	4,294	22,280	10,283
C02	75,411,359	17,873	4,219	24,171	11,034
C03	89,762,950	21,336	4,207	34,884	15,699
C04	78,119,791	18,607	4,198	28,008	11,987
C05	72,374,781	17,061	4,242	27,328	8,098
C06	66,415,506	15,664	4,240	21,720	9,119
C07	73,960,031	17,332	4,267	27,003	11,161
C08	67,666,641	15,658	4,322	24,253	10,660
C09	77,697,774	18,009	4,314	29,670	9,544
Total	1,200,000,000	281,218	4,267	161,262	70,384

### Development of polymorphic SLAF tags and selection of SNP markers

A total of 528,080 SLAF tags were developed from the 300 accessions, with an average depth per sample of 6.27 × (Table S3; Figure S1A). Of these, 238,711 SLAF tags showed polymorphism after all SLAF tags were aligned to the reference genome using the BWA software (Li and Durbin, 2009). The number and distribution of the polymorphic SLAF tags on each chromosome is shown in Table 1. Polymorphic SLAF tags were well-distributed across all chromosomes, with the largest number of SLAF tags (15,699) on chromosome C03, and the fewest SLAF tags (5,236) on chromosome A08.

SNP markers were developed using the sequence with the highest copy number per SLAF as the reference sequence. A total of 1,197,282 SNPs in all accessions were identified, and the integrity of SNPs in the 300 accessions ranged from 80.66 to 97.66% (Figure S2). A further 201,817 highly consistent and confident SNP markers with MAF > 0.05 and integrity >0.8 were obtained (Table S4). These SNP markers covered the whole genome of *B. napus* uniformly (Figure S1B).

By sorting the SNPs in the A- and C-subgenomes (Table 2), more SNPs were found to be distributed in the C subgenome (80,014) than in the A subgenome (63,307). However, due to the larger size of the C subgenome surveyed, the SNP (27 SNPs/100 kb) and gene (15 genes/100 kb) density in the A subgenome was higher than that in the C subgenome, which had 20 SNPs/100 kb and 11 genes/100 kb). The largest number of SNPs (11,515) was located on chromosome C03 (19 SNPs/100 kb), while chromosome A08 had the fewest SNPs (4474) with 24 SNPs/100 kb, and chromosome A10 had the highest SNP density of 31 SNPs/100 kb.

**Table 2:** **Distribution of SNPs and related genes in the ***B. napus*** genome**.

**Chromosome**	**No. of SNPs**	**Chromosome length (Mb)**	**No. of genes**	**No. of SNP per 100 Kb**	**No. of genes per 100 Kb**	***R*^2^-value**
A01	5,756	23.27	3,448	25	15	0.053
A02	6,854	24.79	3,491	28	14	0.091
A03	7,188	29.77	5,476	24	18	0.064
A04	5,261	19.15	2,721	27	14	0.069
A05	7,008	23.07	3,418	30	15	0.060
A06	6,804	24.40	3,741	28	15	0.116
A07	6,411	24.01	3,593	27	15	0.068
A08	4,474	18.96	2,914	24	15	0.096
A09	8,199	33.87	5,157	24	15	0.092
A10	5,352	17.40	2,772	31	16	0.070
C01	9,407	38.83	4,064	24	10	0.282
C02	9,248	46.22	4,411	20	10	0.221
C03	11,515	60.57	7,113	19	12	0.093
C04	11,316	48.93	5,171	23	11	0.240
C05	6,968	43.19	4,895	16	11	0.083
C06	7,134	37.23	4,072	19	11	0.113
C07	9,900	44.77	4,772	22	11	0.252
C08	8,231	38.48	4,614	21	12	0.154
C09	6,295	48.51	5,084	13	10	0.119
A genome	63,307	238.69	36,731	27	15	0.078
C genome	80,014	406.73	44,196	20	11	0.173
AC genome	143,321	645.42	80,927	22	13	0.123

### Identification of SNP hotspots and SNP-rich regions on the genome of *B. napus*

**Figure 1:**
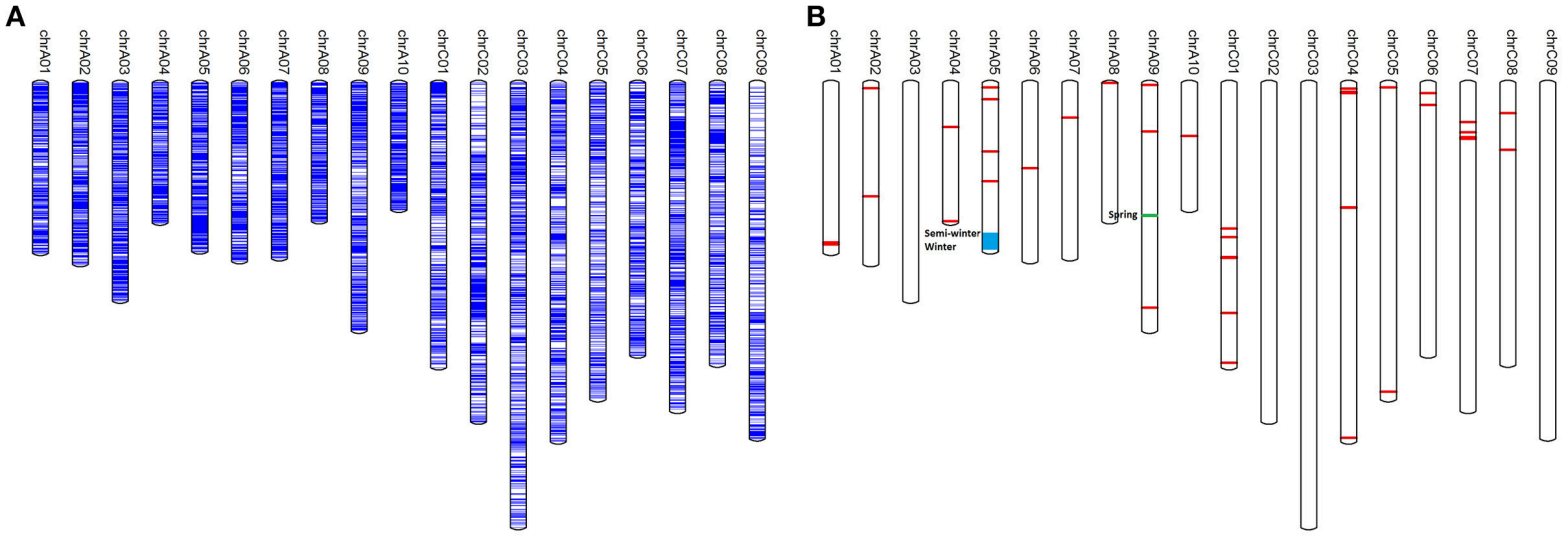
**Distribution of SNP hotspots and SNP-rich regions on chromosomes of ***B. napus***. (A)** The distribution of SNP hotspots on the genome; **(B)** The distribution of SNP-rich regions on the genome in *B. napus*.

A total of 30,877 SNP hotspots (SNP mutation frequency for a specific position >0.8 compared to the reference genome of *B. napus*) were found in the sequenced genome (Table S5), the distribution of which along each chromosome is shown in Figure 1A. The number of SNP hotspots along each chromosome was unequal: the largest number of SNP hotspots (2902) was on chromosome C07, while chromosome A10 had the fewest SNP hotspots (1111). In addition, there were 41 SNP-rich regions containing a total of 4,787 SNPs: these were identified on all chromosomes except for A03, C02, C03 and C09 (Table S6; Figure 1B). A further 100 genes were detected in the SNP-rich regions, where SNPs were distributed upstream, downstream or in intergenic regions relative to these genes (Table S7). From gene ontology (GO) analysis, these genes were involved in response to stress (salt, UV-b, water deprivation, cold, light stimulus, etc.), transcription regulation, defense response to bacteria and fungi, lipid metabolism and transport, hormone synthesis (ethylene, salicylic acid, jasmonic acid, abscisic acid, etc.), vernalization, photomorphogenesis, plant growth development, and regulation (carpel development, seed development, seed germination, pollen tube growth, anther dehiscence, pollen maturation, embryo development ending in seed dormancy, root hair elongation, flower development, anther development etc.) (Figure S3).

In order to detect genomic regions that are potentially differentiated between semi-winter, spring, and winter rapeseeds, SNP-rich regions were assessed for each of the three ecotypes (Figure 1B). Significant differences were observed for two SNP-rich regions on chromosomes A05 and A09 between the three ecotypes (Table S7). The SNP-rich region of *Bna-r-20800000*~*22699999* on A05 was present in winter and semi-winter ecotypes but absent in spring ecotypes, whereas SNP-rich region *Bna-r-30700000*~*30799999* on chromosome A09 only appeared in spring rapeseed ecotypes. In addition, six candidate genes were annotated in the SNP-rich region on chromosome A05, including candidate gene *BnaA05g29990D* (GO: 0010048) involved in the biological process of plant vernalization response. Another candidate gene *BnaA05g33430D* (homologous to *GRF7* of *Arabidopsis thaliana*) participated in floral development. The SNP-rich region on A09 chromosome contained candidate gene *BnaA09g44900D*, which is closely related to plant systemic acquired resistance and defense response. Known QTLs for resistance to *Sclerotinia sclerotiorum* (Wu et al., 2013) and *Leptosphaeria maculans* in oilseed rape (Delourme et al., 2008) were adjacent to this SNP-rich region on chromosome A09.

### Genetic relationships and phylogenetic tree construction

#### Analysis of the genetic relationship among the 300 accessions

Relationship coefficients between the 300 samples were calculated using the 201,817 high-consistency SNPs identified in this study. Of the 45,000 pairwise combinations, 39,278 (87%) had genetic relationship coefficients <0.05 (Figure S4). Hence, there was only very weak or no relationship between accessions in our panel.

#### Phylogenetic tree construction and population principal components analysis

The genotype data for these 201,817 high-quality, polymorphic and single-locus SNPs with MAF > 0.05 in the diversity panel is provided in Table S8, along with the expected chromosome positions of the SNPs on the *B. napus* reference genome (Chalhoub et al., 2014).

**Figure 2:**
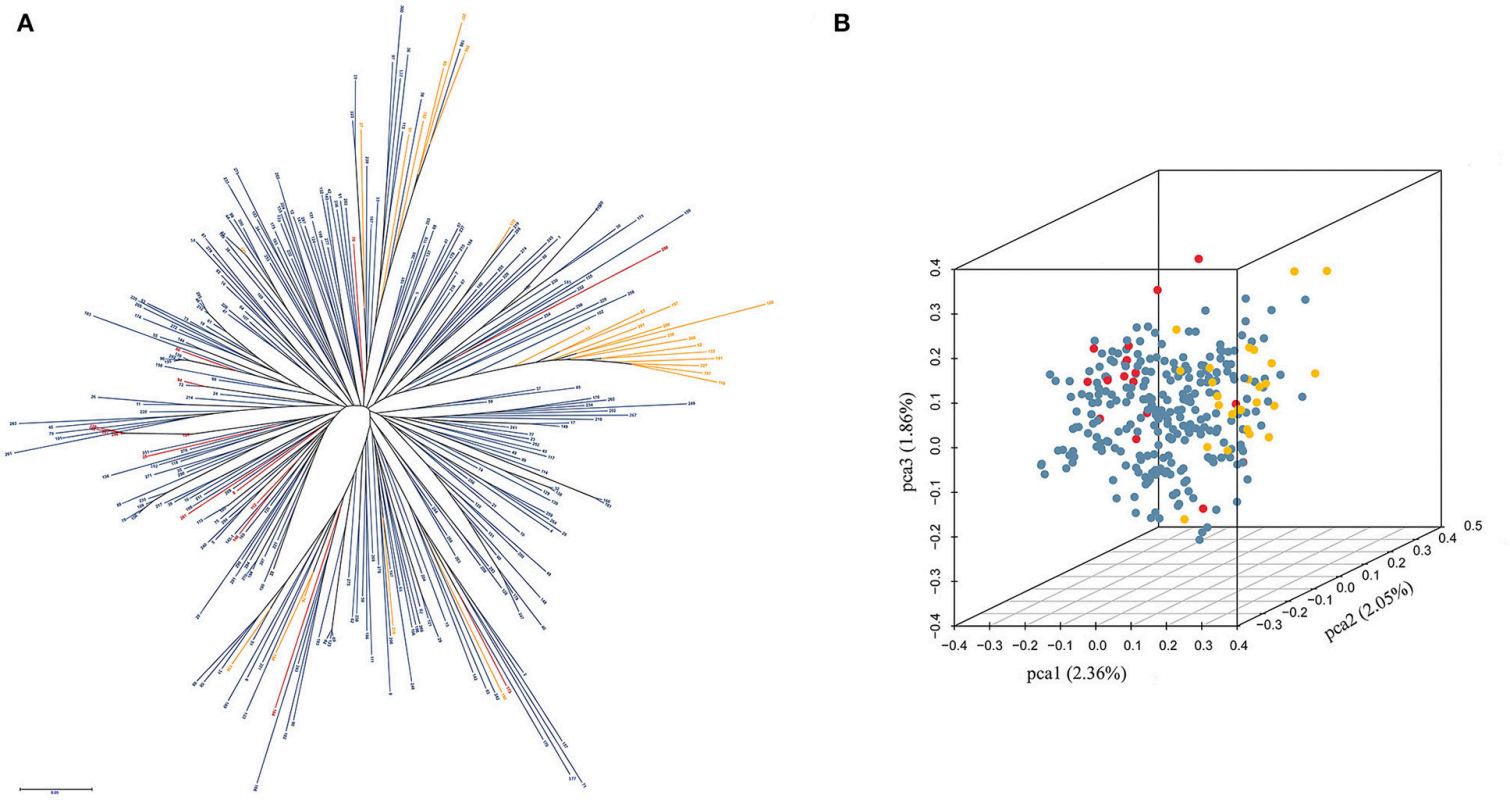
**Clustering and PCA analysis in 300 accessions of ***B. napus***. (A)** Clustering analysis in 300 accessions of *B. napus*; **(B)** PCA analysis in 300 accessions of *B. napus*. The yellow, blue, and red indicate winter, semi-winter, and spring ecotypes of *B. napus*, respectively.

The cluster results showed that most winter rapeseed lines (27) fell into two groups, with only a few clustering into semi-winter groups. In addition, 16 spring accessions were almost all dispersed between the semi-winter groups, suggesting genetic permeation between spring and semi-winter varieties (Figure 2A). Furthermore, the first, second, and the third principal components explained 2.36, 2.05, and 1.86% of the genetic diversity respectively, and the first principal component roughly separated the three ecotypes (Figure 2B).

### Analysis of population structure and linkage disequilibrium (LD)

#### Population genetic structure analysis in *B. napus*

**Figure 3:**
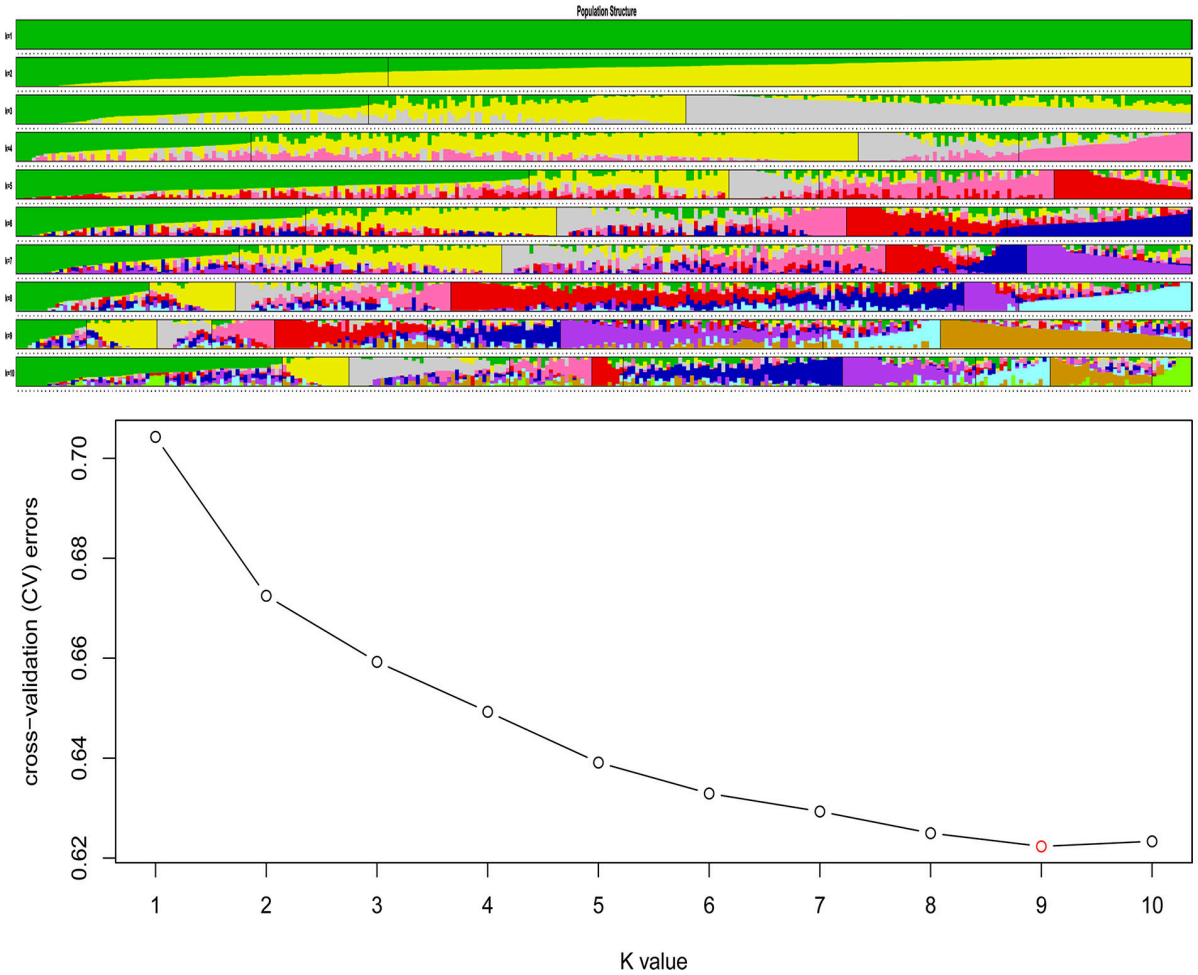
**Population structure of 300 accessions of ***B. napus*****. The accessions were divided into nine subgroups (there was minimum *K*-value when *K* = 9), within each subgroup, the accessions were ordered according to the genetic component, and each line gives the sub-group value, each accession shown as a vertical line partitioned into K colored components represents inferred membership in K genetic clusters.

Population structure as assessed by Admixture (Alexander et al., 2009) suggested an ancestral subgroup number of nine based on cross validation (CV) errors (Figure 3). Of the nine subgroups, the seventh subgroup included the most varieties (67, 22.3%), next to the ninth subgroup (64, 21.3%). Accessions in both groups belonged to the semi-winter ecotype, while most spring rapeseeds clustered into ninth subgroup, and winter-type varieties were mainly concentrated in the second subgroup (Table S9).

#### Patterns of LD across the rapeseed genome

To estimate patterns of LD, SNP linkage along each chromosome was analyzed using an LD decay threshold of *r*^2^ = 0.1. Major differences were observed for different chromosomes, with LD extending from 7.62 Kb (chromosome A02) up to more than 2,000 Kb (chromosomes C01, C02, and C07; Table S10, Figure S5).

**Figure 4:**
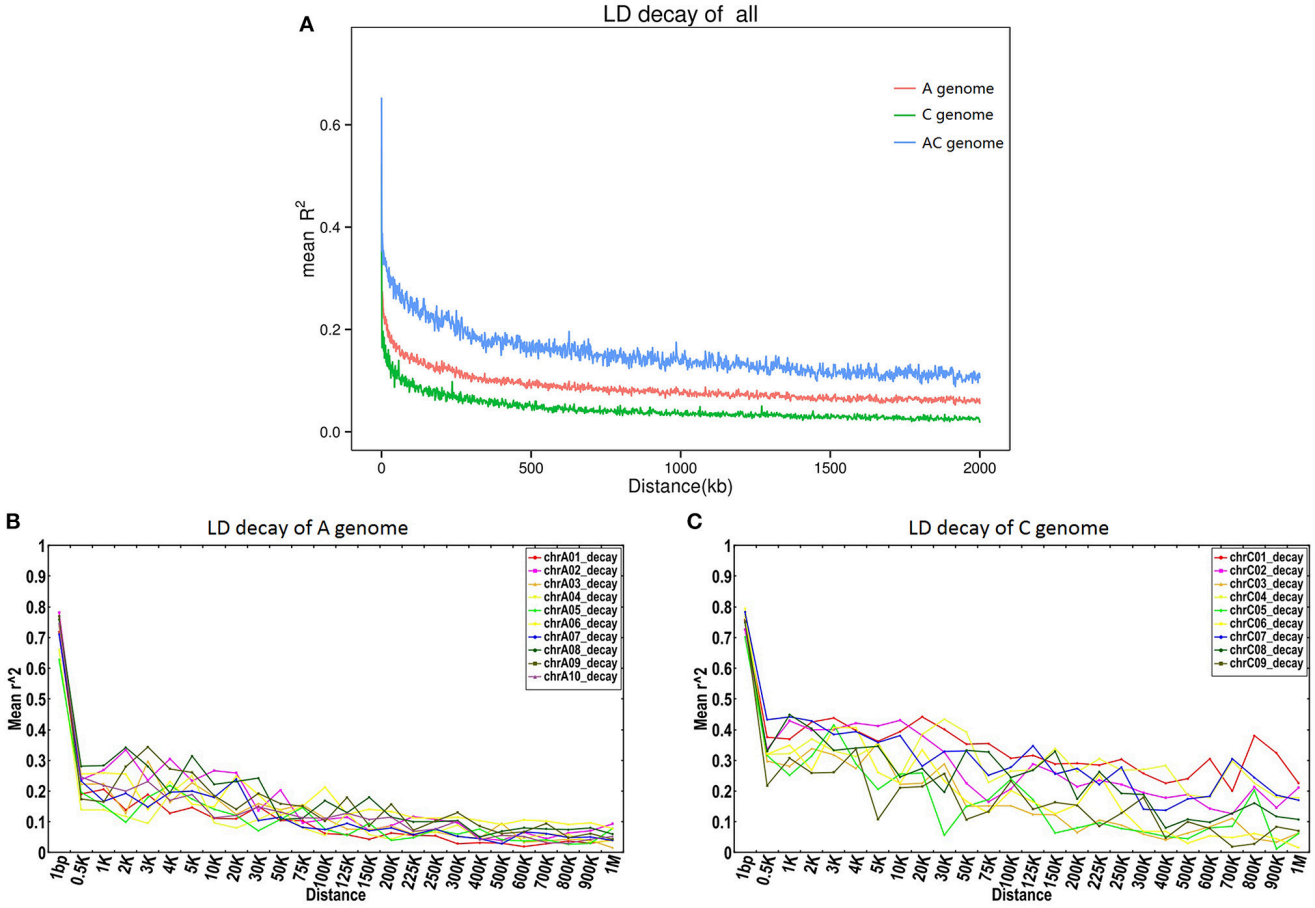
**LD decay on the A and C genomes of ***B. napus***. (A)** LD decay in the A and C genomes; **(B,C)** LD decay curves for each chromosome in the A and C subgenomes respectively.

By comparing the *r*^2^ distribution to the physical distance over the 19 chromosomes, as well as overall across each subgenome, we found that the LD decay (*r*^2^ = 0.1) of the AC genome was 298.95 Kb, while the LD decay was 42.99 Kb and 1,455.28 Kb in the A and C subgenomes respectively (Figure 4).

#### Analysis of blocks based on linkage disequilibrium

The same SNP markers used for LD estimation were used to evaluate the haplotype blocks present in the 300 accessions. A summary of the distribution, size, and number of haplotype blocks along each chromosome is shown in Table 3 and Figure S6. A total of 25,466 conserved haplotype blocks were found in the 300 accessions spanning 80.84 Mb (12.53% of the assembled reference genome). Of these haplotype blocks, 86.54% ranged in size from 0 to 1 Kb, while only 0.34% were >100 Kb in size. In the A subgenome, the mean haplotype block number ranged from 848 (A08) to 1,592 (A09) with an average of 1,208.5, while the mean haplotype block size ranged from 1.65 Mb (A04) to 3.23 Mb (A09) with an average of 2.42 Mb. The mean haplotype block number in the C subgenome ranged from 1,133 (C09) to 2,050 (C03) with an average of 1,487. Haplotype block size in the C subgenome was considerably larger, ranging from 3.34 Mb (C09) to 8.76 (C01) with an average of 6.30 Mb. The percentage of the genome falling into clear haplotype blocks in the A subgenome (10.28%) was also lower than in the C subgenome (14.20%).

**Table 3:** **Distribution of haplotype blocks in the genome of ***B. napus*****.

**Chromosome**	**Chromosome length**	**Block number**	**Block length**	**Frequency (%)**
A01	23,267,856	1,077	1,822,637	7.83
A02	24,793,737	1,240	2,603,566	10.50
A03	29,767,490	1,418	2,825,900	9.49
A04	19,151,660	987	1,649,516	8.61
A05	23,067,598	1,314	2,443,836	10.59
A06	24,396,386	1,320	2,780,562	11.40
A07	24,006,521	1,236	2,122,650	8.84
A08	18,961,941	848	2,060,175	10.86
A09	33,865,340	1,592	3,234,961	9.55
A10	17,398,227	1,053	2,626,566	15.10
C01	38,829,317	1,474	8,763,513	22.57
C02	46,221,804	1,564	7,396,336	16.00
C03	60,573,394	2,050	7,064,423	11.66
C04	48,930,237	1,875	8,356,263	17.08
C05	43,185,227	1,136	3,374,479	7.81
C06	37,225,952	1,199	4,665,746	12.53
C07	44,770,477	1,627	6,404,941	14.31
C08	38,477,087	1,323	7,312,052	19.00
C09	48,508,220	1,133	3,336,424	6.88
Total	645,398,471	25,466	80,844,546	12.53

## Discussion

### Large numbers of SNP markers discovered by SLAF-Seq technology

Genomic data provide researchers novel insight into rapeseed genetic diversity and domestication (Qian et al., 2014; Gazave et al., 2016). In this study, we used 300 rapeseed accessions collected from different regions of China with outgroups from other countries, to sequence genome-wide distributed specific locus amplified fragments (SLAF) for polymorphism detection and genotyping (Sun et al., 2013), with an average sequencing depth of 6.27-fold per accession (>5.0-fold), in order to assure the veracity of the population genetic analyses (He et al., 2011; Han et al., 2016). The mean physical distance between SNP markers was 0.22 Kb, which was dramatically shorter than the mean LD decay distance (298.95 Kb), so the density of SNP markers was sufficient for genetic diversity and association mapping purposes (Morris et al., 2013). Furthermore, the sequenced SNP markers distributed across the entire genome represent most rapeseed genomic regions.

We identified a total of 238,711 polymorphic SLAF tags containing 1,197,282 SNPs, and finally selected 201,817 high-consistency SNPs with MAF > 0.05 and integrity > 0.8. In recent years, SLAF-seq technology has been widely used for high-throughput SNP and InDel marker development, high-density genetic map construction and genome-wide association analyses of important agronomic traits in major crops (Li et al., 2014). Chen et al. (2013) were the first to report first the use of SLAF-seq to develop 89 specific and stable molecular markers in *Thinopyrum elongatum*, which provided a strong case for the application of this new technology. Li et al. (2014) reported a high-density soybean genetic map based on large-scale SNP markers discovered by the SLAF-seq technology, allowing consistent QTLs for isoflavone content across different environments to be identified. Xia et al. (2015) identified 5,142 polymorphic SLAF tags and 148 variants through SLAF-seq technology, and subsequently successfully detected hotspots associated with important agronomic traits in maize. Likewise, Geng et al. (2016) developed 1,933 high quality polymorphic SLAF markers and identified four markers associated with thousand seed weight in rapeseed, as well as a hotspot of ~0.58 Mb on chromosome A09 containing four candidate genes closely associated with seed weight. In sum, previous research has indicated that SLAF-seq technology is a highly efficient method for crop genetic analysis.

In our study, the average SNP distribution density was 22 SNPs/100 Kb, ~3 times the SNP density (6.67 SNPs/100 Kb) of the Illumina Infinium *Brassica* 60K genotyping array (Illumina Inc., San Diego, CA, USA; Clarke et al., 2016). Therefore, the size of blocks we detected was smaller than in previous results (Qian et al., 2014), which facilitates precise haplotype map construction and high-resolution LD analysis (Buckler and Gore, 2007; Gore et al., 2009).

Generally, a haplotype block is a cluster of SNPs (*r*^2^ > 0.8) that tends to travel through the generations as a block (Gabriel et al., 2002; Zondervan and Cardon, 2004). In this study, we found 25,466 conserved haplotype blocks spanning 80.84 Mb (12.53% of the assembled reference genome), most of which ranged in size from 0 to 1 Kb. Qian et al. (2014) detected 3,097 conserved haplotype blocks spanning 182.49 Mb (15.17% of the genome) using 24,994 SNPs from the *Brassica* SNP consortium Illumina Infinium *Brassica* 60K genotyping array (Illumina Inc., San Diego, CA, USA). This study also drew the same conclusion as found in our data, that the number of haplotype blocks in the A subgenome is lower than in the C subgenome. In addition, we found 30,877 SNP hotspots and 41 SNP-rich regions in the *B. napus* genome. There could be several explanations for these. Firstly, there are many regions in the genome that are rich in repetitive sequences, where DNA polymerase errors resulting in strand slippage and inequitable exchange can easily occur (Qin et al., 2015; Clayton et al., 2016). Secondly, mutational hotspot regions often represent recombination hotspots, or vice versa (Mercier et al., 2015). Thirdly, the lower the selective pressure, the greater the accumulation of mutations, and mutated allelic sites in genic regions are usually easily swept away under the relatively greater selective pressure in these regions. Finally, some variable regions result from adaptative pressures, whereby mutations in genes related to adaptive capacity are more likely to be retained, as variability may increase survival probabilities with exposure to environmental stress (Hayward et al., 2015; Weigel and Nordborg, 2015).

### A-subgenome variation is richer than C-subgenome variation in *B. napus* based on population structure and linkage disequilibrium analysis

Semi-winter rapeseed, mainly planted in the Yangtze valley of southern China, switches from vegetative to reproductive growth after a short period of vernalization (Qian et al., 2006). In the past 20 years, the genetic diversity of these three ecotypes of *B. napus* has been widely studied by different molecular marker technologies (Diers and Osborn, 1994; Hasan et al., 2006; Qian et al., 2006, 2014). In our study, the genetic diversity analysis of the three ecotypes did not separate the spring types from the semi-winter types. We propose two main reasons for this related to breeding strategies in China. Firstly, spring rapeseed has the advantage of early maturation, removing seasonal barriers to the oil-rice-rice triple-cropping system in southern China, so genetic exchange between spring type and semi-winter type rapeseed occurred frequently during breeding for early-maturing varieties in this region. Secondly, rapeseed in China has been adapted for planting in spring-type regions such as the Gansu province in the northwest of China, such that genetic components from semi-winter rapeseed have been introgressed into spring types in order to breed new spring rapeseed varieties (Qian et al., 2007).

Special variants can also be selected by ecogeographic adaptation and human selection. It is likely that strong selection for a particular locus controlling one or more agronomic traits may have a large influence on LD and genetic diversity. In genetic experiments in mammals, evolutionary processes are known to drive the selection of individual genetic polymorphisms and haplotype block structure (Guryev et al., 2006). As for the effect of artificial selection on LD in crops, this is thought to mainly reduce the allelic diversity around the major gene loci or QTL responsible for an important agronomic trait such as oil quality, flowering behavior, and biotic or abiotic resistances, with double-low quality oilseed rape a typical example of this effect. With the release of the *B. napus* genome sequence and the development of genome-wide SNPs (Chalhoub et al., 2014), it has become feasible to study LD in rapeseed in depth. Here, we identified whole genome-scale LD patterns in rapeseed and obtained an overall average LD distance of 298.95 Kb. Ecke et al. (2010) analyzed the LD in a population of 85 canola winter rapeseed genotypes using 845 AFLP markers, and found the LD decay distance was about 2~3 cM (1 cM≈500 Kb in *B. napus*). Similar conclusions were drawn by Harper et al. (2012) using associative transcriptomics. However, Xiao et al. (2012) evaluated the extent of LD in a panel of 192 inbred lines of *B*. *napus* worldwide using 451 SSRs, and found that the LD decayed within 0.5–1 cM at the genome level, varying with the population size, genetic background, and genetic drift. Delourme et al. (2013) assessed the extent of LD for spring and winter ecotype oilseed rape, and found LD decayed faster in spring than in winter oilseed rape. The average LD decay distance (*r*^2^ = 0.1) on the A and C subgenomes was also calculated using 24,994 SNP markers in a panel of 203 Chinese semi-winter rapeseed accessions, revealing that mean LD decay was about 10 times faster in the A subgenome (0.25–0.30 Mb) than in the C subgenome (2.00–2.50 Mb; Qian et al., 2014). Overall, the obtained LD decay distance in *B. napus* is about 250–1,500 Kb, which was generally consistent with *Arabidopsis* (~250 Kb; Nordborg et al., 2002), rice (~200 Kb; McNally et al., 2009), soybean (~150 Kb; Lam et al., 2010) and sorghum (~150 Kb; Morris et al., 2013), but higher than the typical cross-pollinated crops like maize (1–10 Kb; Yan et al., 2009). Detailed LD analysis allows us to track down the footprints of domestication and the strong selection bottlenecks associated with cultivation and breeding of *B. napus*.

In the current study, LD decay in the A subgenome was dramatically faster than in the C subgenome, and genetic diversity was higher, indicating that the A subgenome had undergone more recombination. The primary reason for this is thought to be that *B. napus*, originally derived from Europe, underwent frequent crosses with Chinese *B. rapa* to create oilseed varieties suitable for the Chinese climate. Before the 1940s, traditional rapeseed varieties in China were *B. rapa* and *B. juncea* (Fu, 2000), but due to the advantages offered by *B. napus* of high yields, disease-resistance, and extensive adaptability, *B. napus* gradually took the place of the Chinese traditional oilseed rape varieties, and was subsequently planted widely in the Yangtze River Basin in southern China (Liu, 2000). Over 50% of Chinese *B. napus* cultivars are thought to originate from crosses between *B. napus* and *B. rapa* (Qian et al., 2006; Chen et al., 2007). By contrast, it is fairly difficult to carry out *B. napus* × *B. oleracea* crosses successfully (Bennett et al., 2008), which poses a limitation to C genome diversification in *B. napus*. This is also thought to have contributed to the greater LD and lower genetic diversity of the C subgenome relative to the A subgenome in Chinese oilseed rape. In addition, Chalhoub et al. (2014) reported that the C subgenome contains more transposon-rich but recombination-poor regions compared to the A subgenome [transposon-rich regions are often also recombination-poor (Gorelick, 2003), which could also partly explain the significant difference in LD between the A and C subgenomes].

In this study, we developed 201,817 high-confidence SNP markers in a panel of 300 accessions of *B. napus* using SLAF-seq (specific-locus amplified fragment sequencing), of which we found 30,877 SNP “hotspots” and 41 SNP-rich genomic regions, and detected potentially differentiated genomic regions between semi-winter, spring and winter ecotype rapeseed. Subsequent genetic analysis for these 300 accessions validated the breeding history of semi-winter rapeseed, showing introgressions from spring types as well as progenitor species *B. rapa*. Our study provides an important breeding resource, laying the foundation for future analysis of important agronomic traits in *B*. *napus*.

## Author contributions

QZ carried out the genetic analysis and wrote the manuscript and with CZ carried out the genotyping experiments. WZ processed the planting and management for the 300 accessions of *B. napus*. SF and CW made helpful suggestions on the manuscript and paticipated in the development of the population SNP markers. AM critically revised the manuscript. YH and DF provided plant materials, designed, led, and coordinated the overall study. All authors read and approved the final manuscript.

### Conflict of interest statement

The authors declare that the research was conducted in the absence of any commercial or financial relationships that could be construed as a potential conflict of interest.

## Supplementary material

Figure S1 **Distribution of SLAF tags and SNPs on each chromosome of ***B. napus*****. X-coordinate is the length of each chromosome, each yellow stripe stands for a chromosome, and the deeper color indicates a higher density of SLAFs **(A)** or SNPs **(B)** per 1 Mb on the genome of *B. napus*.

Figure S2 **GenTrain score values of SNPs**.

Figure S3 **GO Annotation for genes in SNP-rich regions in ***B. napus*****.

Figure S4 **Analysis of relative kinship in 300 accessions of ***B. napus*****.

Figure S5 **Percentage of block size in the genome of ***B. napus*****.

Figure S6 **Genome-wide distribution of SNPs, LD and related genes in the genome of ***B. napus*****. Concentric circles show structural, functional and evolutionary items of the genome: high LD, SNPs, and related genes in the genome of *B. napus* from inside to outside, respectively.

Table S1 **Information for the 300 inbred lines and assignment to ecotype subgroups of ***B. napus*****.

Table S2 **Sequencing data statistics of the 300 ***B. napus*** accessions**.

Table S3 **SLAF tags statistics of 300 accessions of ***B. napus*****.

Table S4 **SNP statistics of 300 accessions of ***B. napus*****.

Table S5 **SNP hotspots on chromosomes of ***B. napus*****.

Table S6 **SNP-rich regions on chromosomes of ***B. napus*****.

Table S7 **SNP types and associated-gene names in SNP-rich regions on chromosomes of ***B. napus*****.

Table S8 **SNP data of accessions of ***B. napus*****.

Table S9 **Clustering of different-ecotype accessions among subpopulations in ***B. napus*****.

Table S10 **Average distance of linkage disequilibrium (LD) decay on A- and C-subgenome chromosomes of ***B. napus*****.
