# Supplementary figures and images for: Genome-Wide SNP Markers Based on SLAF-Seq Uncover Breeding Traces in Rapeseed (Brassica napus L.)
Source: Front Plant Sci. 2017 Apr 28;8:648. doi: 10.3389/fpls.2017.00648 (PMC5409215; doi:10.3389/fpls.2017.00648)

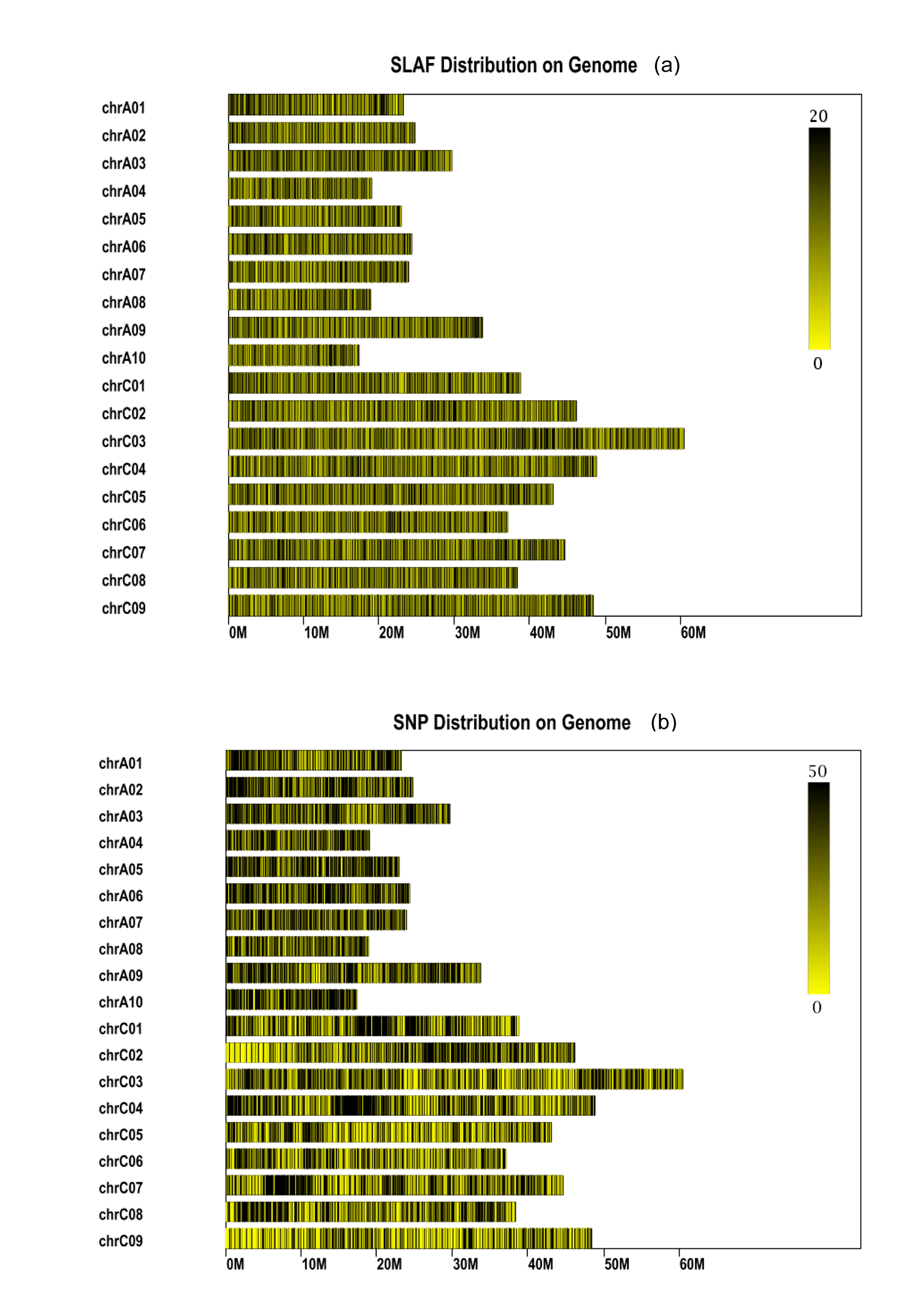

Supplement: Figure S1 — Distribution of SLAF tags and SNPs on each chromosome of B. napus. X-coordinate is the length of each chromosome, each yellow stripe stands for a chromosome, and the deeper color indicates a higher density of SLAFs (A) or SNPs (B) per 1 Mb on the genome of B. napus. [file Image1.TIF]

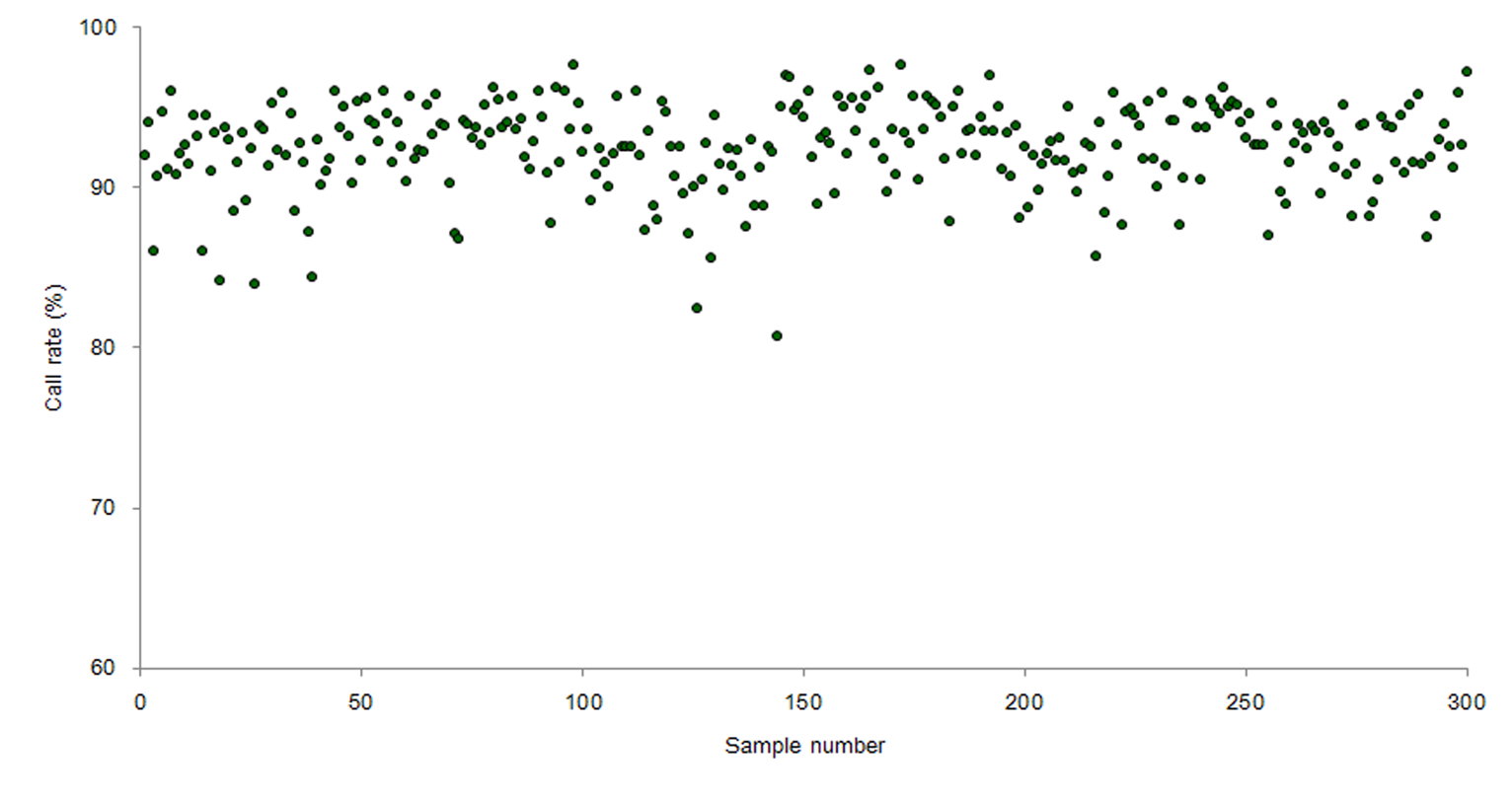

Supplement: Figure S2 — GenTrain score values of SNPs. [file Image2.TIF]

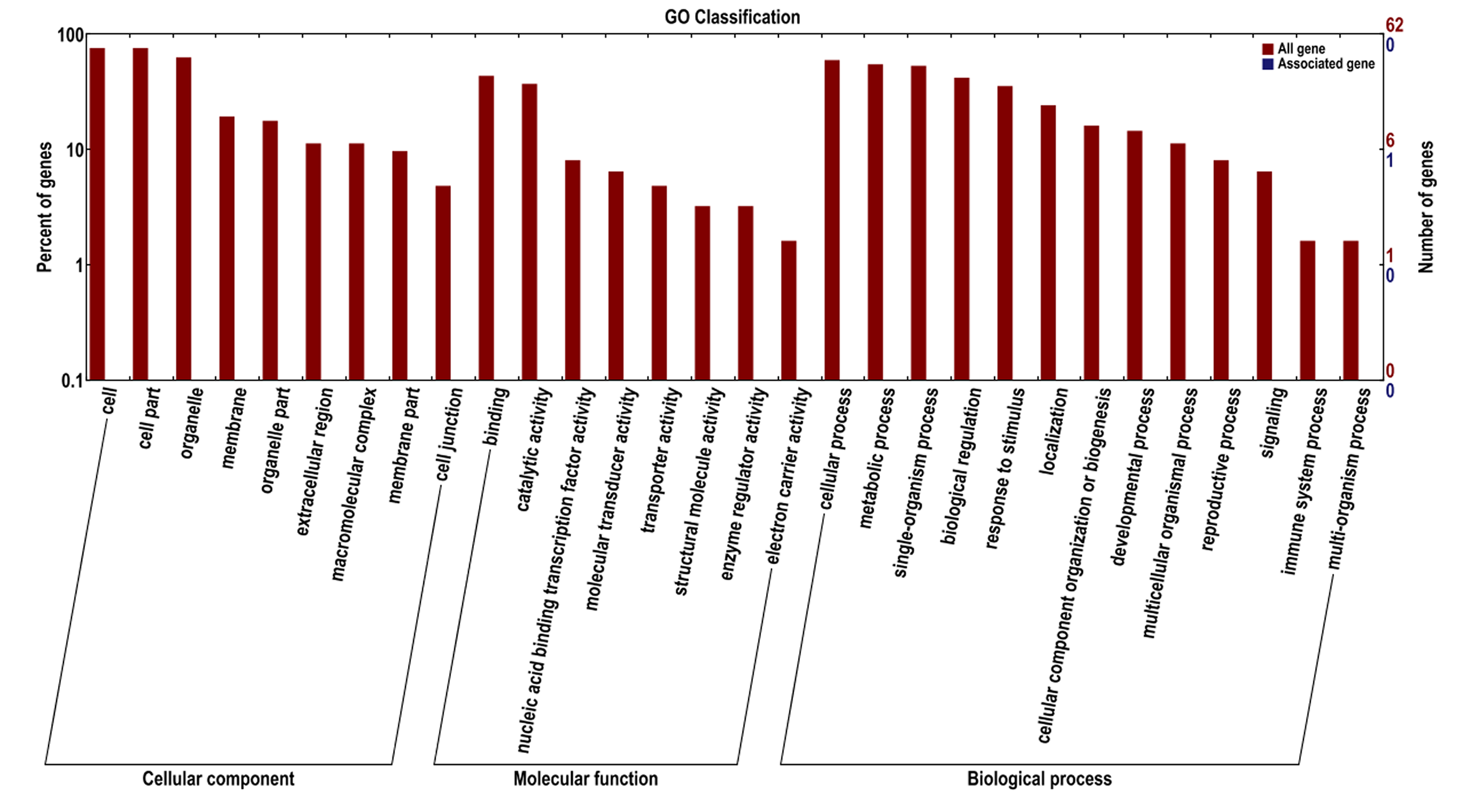

Supplement: Figure S3 — GO Annotation for genes in SNP-rich regions in B. napus. [file Image3.TIF]

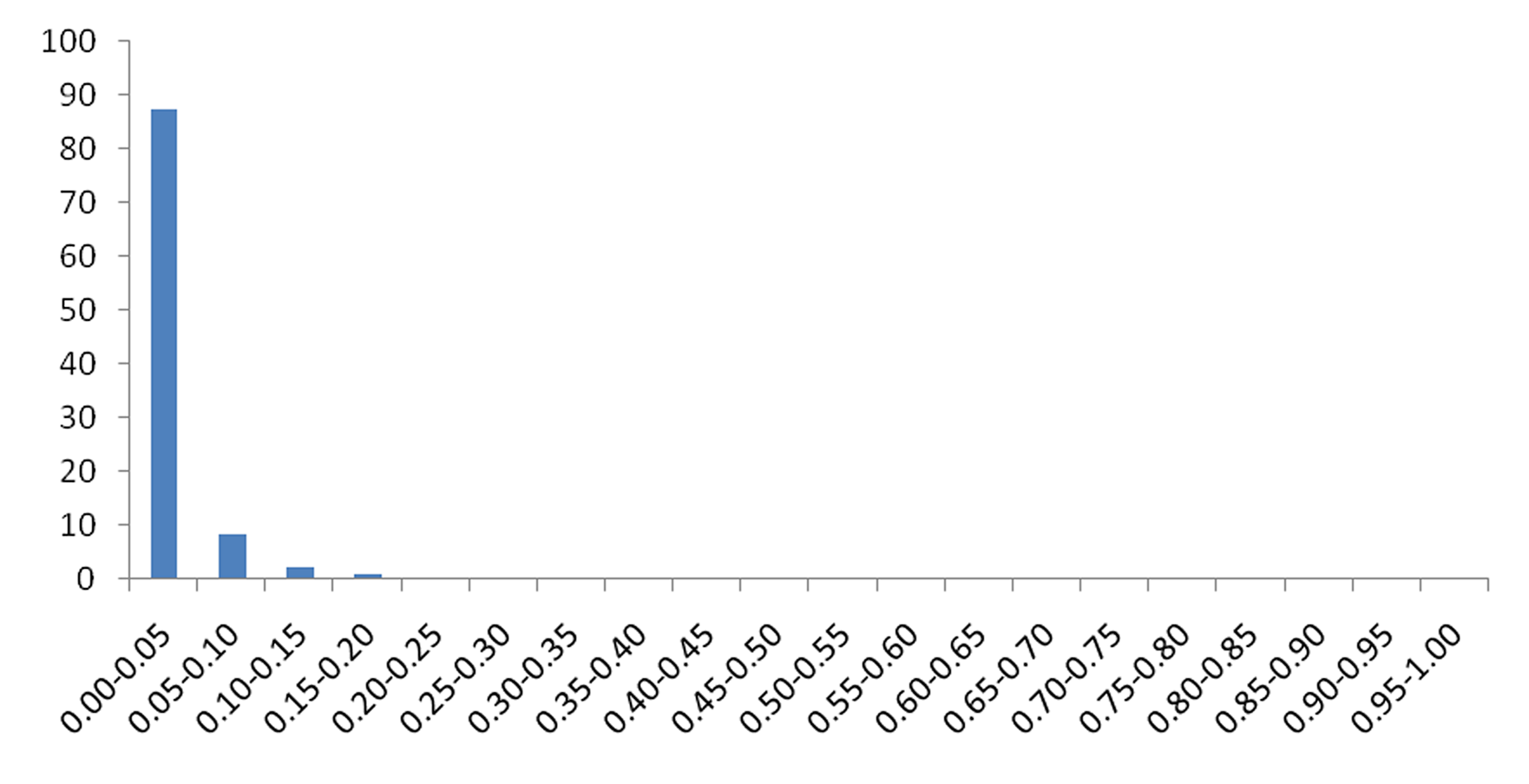

Supplement: Figure S4 — Analysis of relative kinship in 300 accessions of B. napus. [file Image4.TIF]

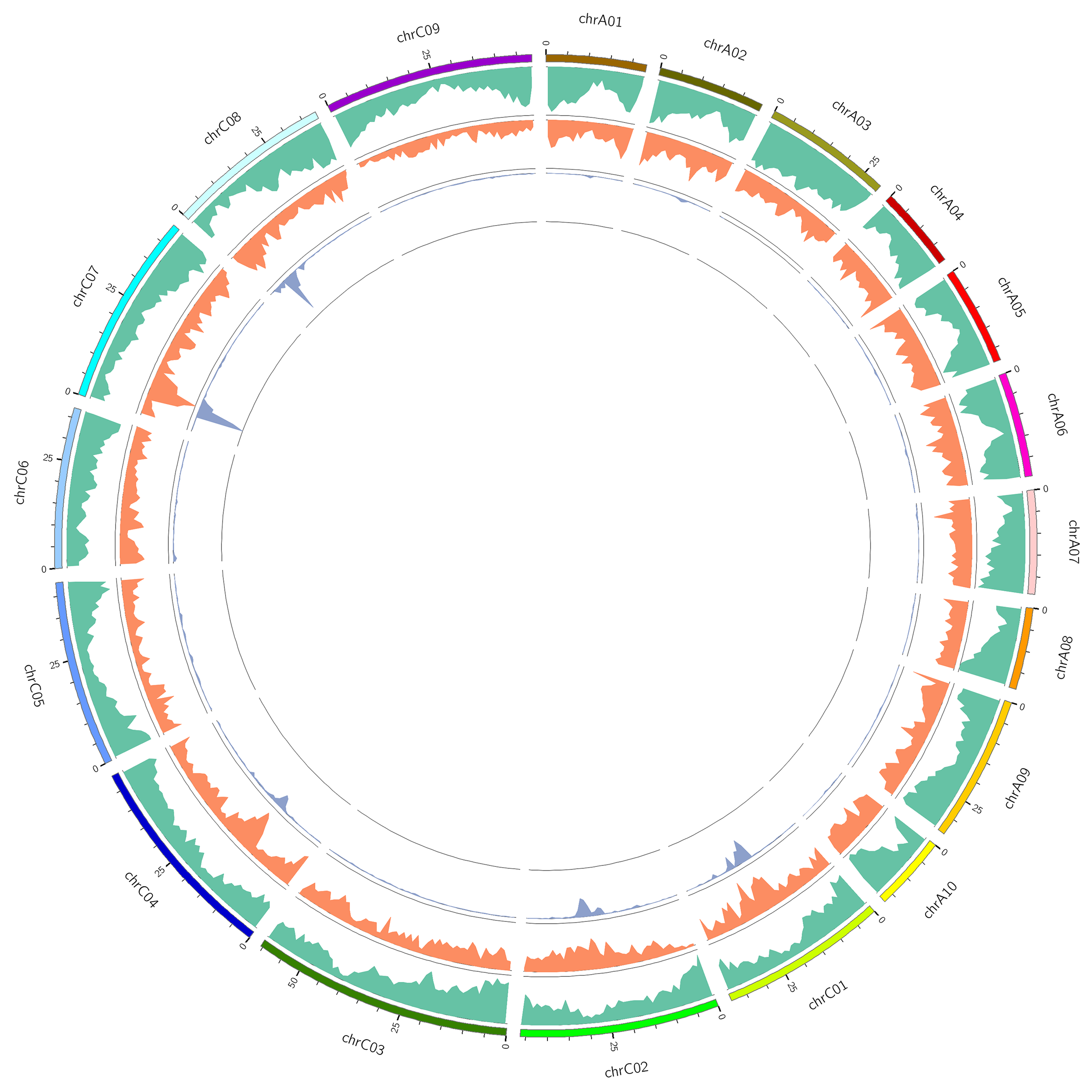

Supplement: Figure S5 — Percentage of block size in the genome of B. napus. [file Image5.TIF]

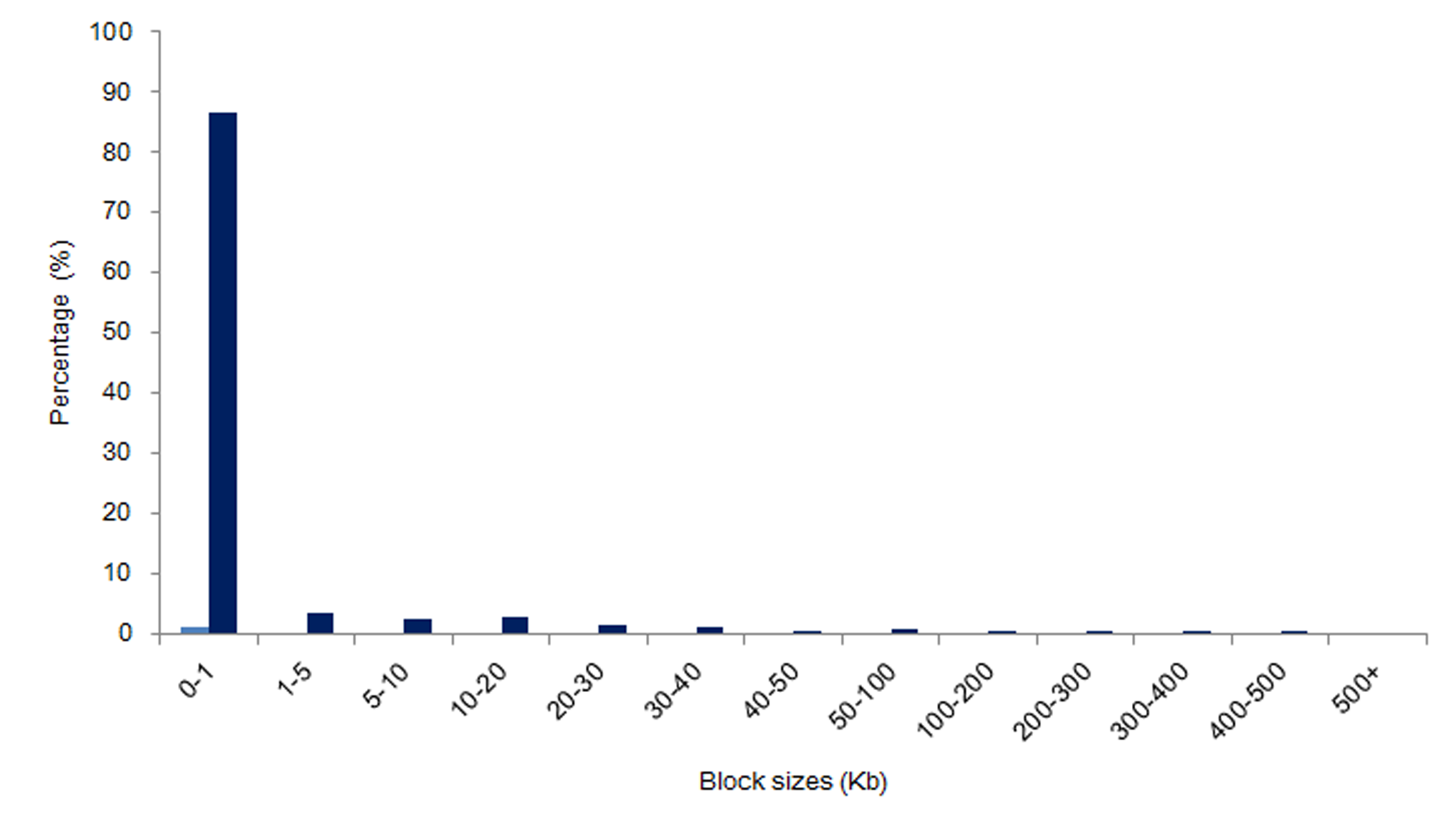

Supplement: Figure S6 — Genome-wide distribution of SNPs, LD and related genes in the genome of B. napus. Concentric circles show structural, functional and evolutionary items of the genome: high LD, SNPs, and related genes in the genome of B. napus from inside to outside, respectively. [file Image6.TIF]
