# Supplementary material for: Genome-Wide SNP Markers Based on SLAF-Seq Uncover Breeding Traces in Rapeseed (Brassica napus L.)
Source: Front Plant Sci. 2017 Apr 28;8:648. doi: 10.3389/fpls.2017.00648 (PMC5409215; doi:10.3389/fpls.2017.00648)
Supplement: Table S10 — Average distance of linkage disequilibrium (LD) decay on A- and C-subgenome chromosomes of B. napus. [file Table10.DOC]

**Table S10 Average distance of linkage disequilibrium (LD) decay on A- and C- sub-genome chromosomes**

| Chromosome | *R^2^*_half value | *R^2^*_half_Decay_Value | Decay Value (*R^2^*=0.1) |
| --- | --- | --- | --- |
| chrA01 | 0.305086234 | 0.647700036 | 20.91487143 |
| chrA02 | 0.344415189 | 0.771472828 | 106.4146267 |
| chrA03 | 0.322849716 | 0.710910573 | 9.279366845 |
| chrA04 | 0.278843343 | 0.437744882 | 19.08697965 |
| chrA05 | 0.264171714 | 0.597808186 | 21.22801557 |
| chrA06 | 0.327016179 | 0.766542266 | 141.2645522 |
| chrA07 | 0.312223116 | 0.64798457 | 27.8063829 |
| chrA08 | 0.323446173 | 0.783284211 | 61.80201266 |
| chrA09 | 0.313638681 | 0.53789009 | 95.02232162 |
| chrA10 | 0.291036008 | 0.762404445 | 25.59540396 |
| chrC01 | 0.366499104 | 1.104997941 | NA |
| chrC02 | 0.361013027 | 1.633045597 | NA |
| chrC03 | 0.353705635 | 0.964485643 | 112.3157352 |
| chrC04 | 0.351391263 | 1.018532785 | 1993.290052 |
| chrC05 | 0.350341186 | 0.850712193 | 63.07905847 |
| chrC06 | 0.376737028 | 0.973905983 | 177.3662239 |
| chrC07 | 0.372155728 | 16.37445225 | NA |
| chrC08 | 0.356265391 | 3.018352178 | 399.8513717 |
| chrC09 | 0.34738762 | 0.989181351 | 66.73756528 |
| all A | 0.293486806 | 0.626988902 | 42.99235196 |
| All C | 0.359209933 | 1.096259366 | 1455.283703 |
| All AC | 0.310896629 | 0.796373295 | 298.9481999 |

Note: NA means not be applicable.
